# Supplementary material for: Inhibition of Human Osteoclast Differentiation by Kynurenine through the Aryl-Hydrocarbon Receptor Pathway
Source: Cells. 2021 Dec 10;10(12):3498. doi: 10.3390/cells10123498 (PMC8700497; doi:10.3390/cells10123498)
Supplement: Supplementary file 1 [file cells-10-03498-s001.zip › cells-1413967-supplementary.pdf]

**SUPPLEMENTARY DATA**

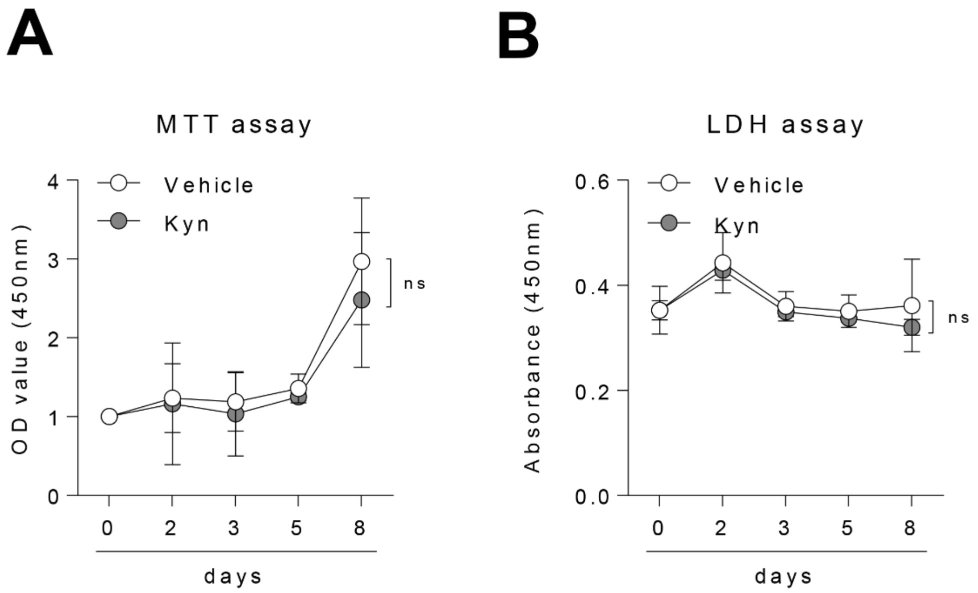


**Figure S1.** Kyn does not affect cell viability. Human PBMC CD14+ cells were cultured with Kyn (100 μM) under osteoclast differentiation conditions. On the indicated time, absorbance at 450 nm was measured and Data are shown as mean ± SD of three independent experiments and a one-way ANOVA test was applied.

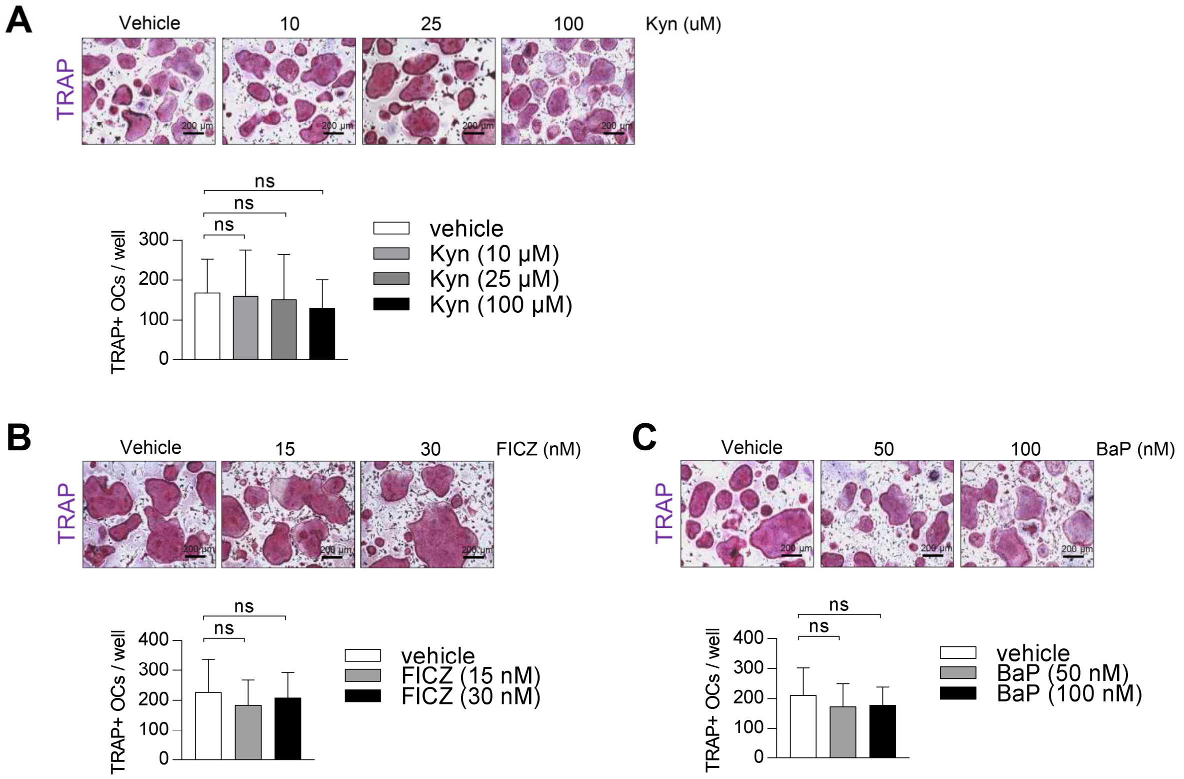


**Figure S2.** AhR agonists have no effect on osteoclasts derived from BMMs. BMMs were isolated from femurs and tibiae of C57BL/6 mice. The cells were cultured with (**A**) Kyn (0, 10, 25, 100 μM), (**B**) FICZ (0, 15, 30 nM) or (**C**) BaP (0, 50, 100 nM) for 4 days with murine M-CSF (50 ng/mL) and murine RANKL (100 ng/mL). The cells were stained with TRAP on day 4, and the TRAP+ osteoclasts were counted (more than ten nuclei). Data are shown as mean ± SD of more than three independent experiments and a one-way ANOVA test was applied.
